# Malignant rhabdoid tumor in a solitary kidney arising in an adult patient with chronic obstructive renal calculi

**DOI:** 10.1016/j.ijscr.2019.04.021

**Published:** 2019-04-16

**Authors:** Y. Ayari, S. Ben Rhouma, H. Boussaffa, M. Krarti, L. Charfi, M. Jrad, Y. Nouira

**Affiliations:** a Department of Urology, La Rabta University Hospital, Tunis, Tunisia; b Department of Anatomopathology, Institute Salah-Azaïz, Tunis, Tunisia; c Department of Radiology, La Rabta University Hospital, Tunis, Tunisia

**Keywords:** Renal, Malignant, Rhabdoid tumor, Adult, Hydronephrosis, Renal calculi

## Abstract

**Introduction:**

Malignant rhabdoid tumor (MRT) is one of the most aggressive and lethal malignancies in pediatric oncology, MRT of the kidney is an uncommon renal tumor in children and it’s extremely rare in adult patients. With only seven previously reported adult cases in the English-literature, to our knowledge this is the first case that is associated with renal calculi.

**Presentation of case:**

We present the case of a 65-year-old man with an MRT arising in a solitary kidney with multiple enlarged lymph nodes that compressing the inferior vena cava.

**Discussion:**

Malignant rhabdoid tumor of the kidney was originally described as a “rhabdomyosarcomatoid” variant of Wilm’s tumor due to the resemblance of cells to rhabdomyoblasts, now this type of tumor is recognized as distant and unique malignant renal tumor. It affects usually children before the age of 2 years. Tumor tissue sampling is required to make the diagnosis of MRTK, based on either nephrectomy, core biopsy, or autopsy specimens. There is no established standard of care due to the paucity of cases. Surgery is considered to be the first choice of treatment if possible.

**Conclusion:**

This case report reinforces the importance of recognizing this entity in the adult population, and discuss the possible treatment options of this rare and highly aggressive tumor.

## Highlights

• Malignant rhabdoid tumor of the kidney in adult patients are extremely rare and have the worst prognosis of all renal tumors.
• The histological features and immunohistochemical staining may aid in confirming the diagnosis of these tumors.
• It is necessary to consider this entity in front of renal masses when such type of renal tumor is encountered in adult patients.

## 1 Introduction

Malignant rhabdoid tumors of the kidney (MRTK) are uncommon renal tumors which mainly occur in children and are extremely rare in adult patients. MRTK is a highly aggressive neoplasm with a short survival time after its diagnosis, and it is characterized by the early onset of local and distant metastases and resistance to chemotherapy. Here we describe another adult case of renal MRTK associated with renal calculi, identified in a patient’s solitary right kidney; the diagnosis was established a CT- guided core biopsy with multiple needle passes, and the final diagnosis was made based on characteristic histological findings and immunohistochemical features of this kind of tumors. Our work has been reported in line with the SCARE criteria [1].

## 2 Presentation of case

**Fig. 1:**
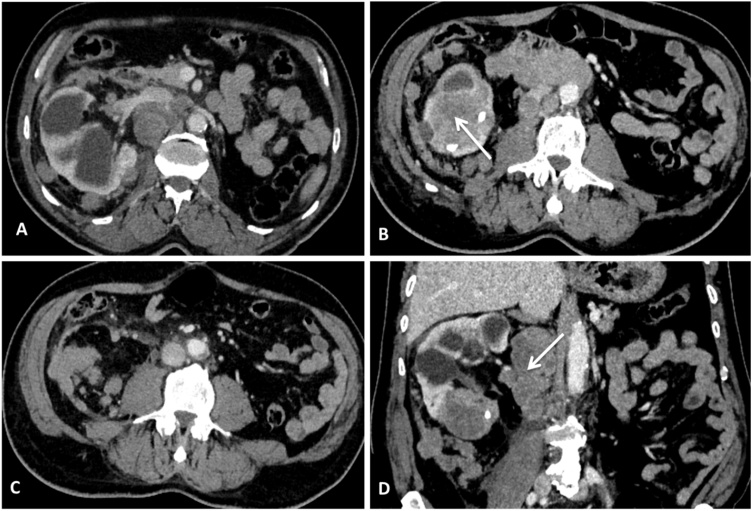
CT scan showing **(A):** hydronephrotic solitary right kidney contiguous nodules in the perirenal fat **(B):** a heterogeneous mass in the lower pole of the kidney (arrow) with renal calculi **(C)** multiple nodules in the perirenal fat (**D):** a coronal plane showing the mass and the voluminous lymph node compressing the inferior vena cava (arrow).

A 65-year-old man with history of hypertension, and long-standing history of renal calculi, requiring left nephrectomy for hydronephrotic kidney twenty years ago, repetitive right percutaneous nephrolithotomy that occurred more than two years ago followed by extracorporeal shock wave lithotripsy. The patient was incompliant to regular urological follow up. Upon his most recent presentation, he complained of right flank pain with progressive fatigue, a weight loss of 5 kg, and abdominal distention that started one month ago, there was no fever or gross hematuria. Physical examination revealed normal vital signs, moderate tenderness in the right costovertebral angle and lower abdominal right quadrant, with lower extremity edema. No abnormalities were noted on relative blood tests (blood Creatinine was 19 mg/L). Ultrasonography revealed a hydronephrotic kidney with multiple calculi and the presence of a mass in the lower pole of the kidney with unclear boundary. An abdominal CT scan with contrast was performed, revealed an enlarged right kidney with marked hydronephrosis with lower calyceal stones less than 10 mm in maximum dimension, there was an ill-defined heterogeneous mass of about 53 × 56 mm in the lower pole of the kidney, increased density in the perirenal fat with contiguous sub capsular nodules with the same semiological characteristics of the mass. There were voluminous lymph nodes along the para aortic region that compressing the inferior vena cava (Fig. 1).

**Fig. 2:**
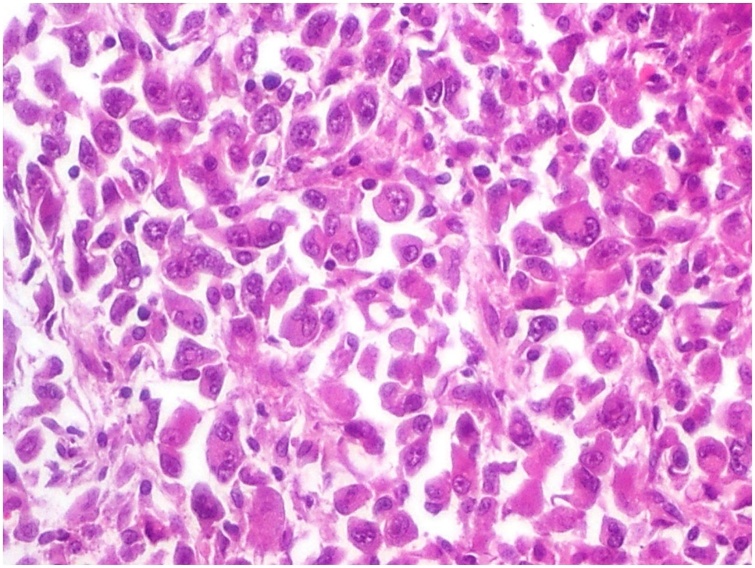
Histological features showing non cohesive tumor cells, large and round to ovoid shapes with abundant eosinophilic cytoplasm, vesicular nuclei, prominent nucleoli, and certain cells had eccentric nuclei.

The patient underwent a CT-guided core biopsy of the renal mass, we performed 5 needle passes and obtained large biopsy specimens. Microscopically, the tumor cells were non cohesive, large and round to ovoid shapes, with abundant eosinophilic cytoplasm, vesicular nuclei, prominent nucleoli, certain tumor cells had eccentric nuclei, and certain areas of the tumor exhibited necrosis in all the biopsy specimens (Fig. 2).

Immunohistochemically, the tumor cells were strongly positive for vimentin, the staining was negative for myoglobin, desmin, or muscle type actin. Accordingly, a pathological diagnosis of pure MRTK was established based on the microscopic features and immunohistochemical findings. The patient’s condition continued to deteriorate and he died few days after the diagnosis was established and before any therapeutic decision.

## 3 Discussion

Malignant rhabdoid tumor of the kidney was originally described as a “rhabdomyosarcomatoid” variant of Wilm’s tumor due to the resemblance of cells to rhabdomyoblasts [2]. Subsequent studies failed to confirm myogenous differentiation, and now this type of tumor is recognized as distant and unique malignant renal tumor. It affects usually children before the age of 2 years (11–18 months) [6], for adult patients with MRTK; the age of diagnosis has ranged from 32 to 60 years, with no predominant gender. The tumor is associated with uniformly aggressive behavior and a poor prognosis. Since MRTK was originally described, malignant rhabdoid tumors was originally reported in practically every location in the body including central nervous system, liver, soft tissues, lung, skin, heart and urogenital system such as the bladder or prostate [3]. The extra renal tumors can be associated with other malignancies (composite rhabdoid tumors) or may constitute the only component (pure rhabdoid tumor) [4]. Several carcinomas arising in the kidney have been observed to have rhabdoid features, including renal clear cell, papillary, transitional cell, collecting duct, and chromophobe carcinomas [5].

Patients with MRTK typically exhibit an abdominal mass, rarely hematuria and abdominal or flank pain. Symptoms arising from metastases are common at diagnosis, which mainly affect the lungs, liver, brain, and less commonly bone. In a small subset of patients, the tumor is discovered incidentally in radiological examinations. There are no characteristic findings of imaging studies aids in distinguishing MRTK in adult patients, whereas CT scan or MRI findings suggesting rhabdoid tumors of kidney including a large lobulated and heterogeneous mass, usually located centrally within the kidney, with calcifications and sub capsular fluid accumulation, are often observed in children [7]. Tumor tissue sampling is required to make the diagnosis of MRTK, based on either nephrectomy, core biopsy, or autopsy specimens. On microscopic examination, the classic appearance is that all tumors contained non cohesive cells, with prominent nucleoli, eccentric vesicular nuclei, and fibrillar eosinophilic cytoplasmic inclusions, mitoses are frequent and necrosis is common. Immunohistochemically the tumor cells are frequently stained diffusely positive for vimentin and most are positive for epithelial membrane antigen and/or cytokeratine. Positivity for neuron-specific enolase, smooth muscle actin, glial fibrillary acidic protein, and CD 99 has rarely been seen in MRTK. The loss of INI-1 protein nuclear expression on immunohistochemical exam is one of the essential criteria for pathologic diagnosis of MRT [8], and it is helpful to differentiate the pure MRTK from other renal neoplasms with rhabdoid differentiation [9].

There is no established standard of care due to the paucity of cases. Surgery is considered to be the first choice of treatment if possible [10]. After the primary tumor is surgically removed, the current adjuvant treatment involves an aggressive multimodal approach with a combination of radiation therapy and various chemotherapy regimens that typically included cisplatin, vincristine, ifosfamide, etoposide, cyclophosphamide, and methotrexate [11]. MRTK have the worst prognosis of all renal tumors. It is highly aggressive with a short survival time and metastases early, with up to 80% of patients presenting with metastatic disease [4].

## 4 Conclusion

Malignant rhabdoid tumor of the kidney in adult patients are extremely rare and have the worst prognosis of all renal tumors, the histological features and immunohistochemical staining may aid in confirming the diagnosis of these tumors. It is necessary to consider this entity in front of renal masses when such type of renal tumor is encountered in adult patients. In addition, further studies and larger case series are required to identify the ideal post-operative protocol for these aggressive tumors.

## Conflicts of interest

The authors declare that there is no conflict of interest regarding the publication of this paper.

## Sources of funding

No source of funding.

## Ethical approval

La Rabta University Hospital ethic committee, Tunis, Tunisia.

## Consent

Written informed consent was obtained from the patient for publication of this case report and accompanying images.

## Author contribution

**Ayari Y;** concept, design, data collection, data analysis, interpretation, and writing the paper.

**Ben Rhouma S;** concept, design, data collection, data analysis, interpretation, and writing the paper.

**Boussaffa H;** data collection, data analysis, and interpretation.

**Krarti M;** data collection.

**Charfi L;** performed the histological examination.

**Jrad M;** performed the CT-guided core biopsy, interpretation.

**Nouira Y;** writing the paper.

## Registration of research studies

This is no research study.

## Guarantor

Ayari Yassine.

## Provenance and peer review

Not commissioned, externally peer-reviewed.
